# Corrosion Sensors for Structural Health Monitoring of Oil and Natural Gas Infrastructure: A Review

**DOI:** 10.3390/s19183964

**Published:** 2019-09-13

**Authors:** Ruishu F. Wright, Ping Lu, Jagannath Devkota, Fei Lu, Margaret Ziomek-Moroz, Paul R. Ohodnicki

**Affiliations:** 1 National Energy Technology Laboratory, Pittsburgh, PA 15236, USA; 2 Leidos Research Support Team, Pittsburgh, PA 15236, USA; 3 National Energy Technology Laboratory, Albany, OR 97321, USA

**Keywords:** corrosion sensors, oil and gas industry, optical fiber sensors, distributed chemical sensing, passive RFID sensors, surface acoustic wave sensors, structural health monitoring

## Abstract

Corrosion has been a great concern in the oil and natural gas industry costing billions of dollars annually in the U.S. The ability to monitor corrosion online before structural integrity is compromised can have a significant impact on preventing catastrophic events resulting from corrosion. This article critically reviews conventional corrosion sensors and emerging sensor technologies in terms of sensing principles, sensor designs, advantages, and limitations. Conventional corrosion sensors encompass corrosion coupons, electrical resistance probes, electrochemical sensors, ultrasonic testing sensors, magnetic flux leakage sensors, electromagnetic sensors, and in-line inspection tools. Emerging sensor technologies highlight optical fiber sensors (point, quasi-distributed, distributed) and passive wireless sensors such as passive radio-frequency identification sensors and surface acoustic wave sensors. Emerging sensors show great potential in continuous real-time in-situ monitoring of oil and natural gas infrastructure. Distributed chemical sensing is emphasized based on recent studies as a promising method to detect early corrosion onset and monitor corrosive environments for corrosion mitigation management. Additionally, challenges are discussed including durability and stability in extreme and harsh conditions such as high temperature high pressure in subsurface wellbores.

## 1. Introduction

### 1.1. Corrosion Problems in Oil and Natural Gas Industry

Corrosion has been a great concern in the oil and natural gas (O&G) industry because it adversely affects infrastructure in exploration, production, processing, and transport with significant economic costs and safety considerations [1,2,3]. For domestic O&G exploration and production in the U.S., direct corrosion costs were determined to be about $1.4 billion annually, according to a study released in 2002, with $589 million attributed to surface piping and facility costs, $463 million to downhole tubing expenses, and $320 million to capital expenditures related to corrosion [4]. There are more than 528,000 km (328,000 miles) of natural gas transmission and gathering pipelines, and 119,000 km (74,000 miles) of crude oil transmission and gathering pipelines. The estimated corrosion-related cost is about $5.8 billion annually to monitor, replace, and maintain these assets [4]. According to the Pipeline and Hazardous Materials Safety Administration (PHMSA) database, corrosion has caused ~25% of the natural gas transmission and gathering pipeline incidents over the last 30 years, and 61% out of corrosion caused incidents were due to internal corrosion [5,6]. It has been challenging to monitor internal corrosion effectively as the inside of pipeline is not readily accessible during regular maintenance and inspection. Moreover, corrosion can occur at some random locations inside and outside pipelines over thousands of miles. Therefore, it is of crucial importance to locate corrosion events along the long-distance infrastructure for effective real-time corrosion monitoring.

Corrosion is an electrochemical process involving oxidation of metallic materials, causing mass loss and structural deterioration. An electrochemical system is essentially composed of an anode, a cathode, and an electrolyte. The anode is the corroding site on the steel, Reaction 1, and the cathode is where the reduction reaction occurs. The electrolyte is usually an aqueous solution with dissolved salts (e.g., NaCl) and corrosive species, and it connects the anode and cathode through ionic conductivity so that the electron transfer can be balanced between the two electrodes. In the anaerobic subsurface wellbores, ubiquitous acidic gases CO_2_ and H_2_S can dissolve into the electrolyte, reduce the pH and promote cathodic reactions through Reactions 2–4, accelerating corrosion process [7,8,9,10,11,12]. Although most downhole hydrocarbon reservoirs have virtually no dissolved oxygen in the fluids, the presence of dissolved O_2_ in drilling fluid can be a major concern for corrosion of drill pipelines and well casing as O_2_ is a strong oxidant even at ppb or ppm levels, Reaction 5 [2,13,14,15].

Anode:  Fe → Fe^2+^ + 2 e^−^                (1)

Cathode:  2 CO_2_(aq) + 2 H_2_O(l) + 2 e^−^ → H_2_(g) + 2 HCO_3_^−^(aq) (2)

2 H_2_S(aq) + 2 e^−^ → H_2_(g) + 2 HS^−^(aq) (3)

2 H^+^(aq) + 2 e^−^ → H_2_(g) (4)

0.5 O_2_(aq) + H_2_O(l) + 2 e^−^ → 2 OH^−^(aq) (5)

Besides mass loss due to electrochemical reactions, corrosion combined with mechanical effects can cause undesirable cracking and resulted catastrophic failures during oil and gas exploration, drilling, production, processing, and transport due to hydrogen induced cracking (HIC), sulfide stress cracking (SSC), stress corrosion cracking (SCC), and corrosion fatigue (CF) [16]. In this scenario, localized corrosion and pitting caused by H_2_S or Cl^−^ are particularly detrimental as a structural weak point is forming and can lead to cracking even when the external force is still within the rated mechanical stress. Microbes such as sulfate-reducing bacteria can also promote corrosion through producing H_2_S [15,17].

As a thermodynamically favorable process, corrosion is difficult to prevent, but can be kinetically controlled through corrosion mitigation and protection. Real-time in-situ monitoring of corrosion and associated parameters facilitates structural health evaluation and effective mitigation strategies, improving infrastructure security and reducing cost caused by catastrophic failures.

### 1.2. Functions and Categories of Corrosion Sensors

Implementing the best available corrosion prevention and control practices could save 25–30% of annual corrosion costs in the U.S. [4]. Effective corrosion monitoring bolsters the corrosion management systems and informs the decision-making entities. Monitoring corrosion rates enables service life evaluation and guides maintenance management. Carbon steel is commonly used in the O&G industry. For example, it is used for transmission pipes, drill pipes, and casing tubing [18], because of its mechanical properties and economic cost. However, it is prone to corrosion in service environments. Real-time corrosion monitoring and proper mitigation/maintenance are critical to maintaining the corrosion rate within an acceptable range to ensure that the infrastructures (e.g., pipes) meet the designed service life. In-situ and online monitoring of the early onset of corrosion also allows corrosion-related structural health monitoring (SHM) by recognizing early signs of structural risks, such as localized corrosion and micro-cracking, before structural failures and catastrophic events resulting from corrosion can occur. Furthermore, in-situ monitoring of corrosive environments facilitates corrosion mitigation strategies by identifying corrosion causes such as water, pH, Cl^−^, CO_2_ and H_2_S. Besides monitoring corrosion rates, locating the corrosion spots or localized corrosion is also of significant value for further inspection and effective mitigation, especially for thousands of miles of transmission pipelines. Additionally, for the O&G industry applications, high durability and stability are required for corrosion sensors in extreme service conditions such as high temperature high pressure (HTHP) during drilling and production up to 200+ °C and 100 MPa [18].

**Figure 1:**
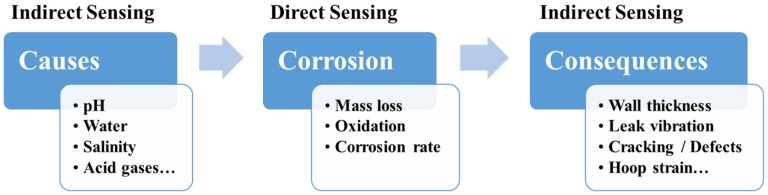
Two categories of corrosion sensors (direct and indirect) to monitor corrosion process from causes to consequences.

Numerous corrosion sensor technologies have been developed based on different sensing principles for different types of corrosion. They can be generally categorized into two types: direct and indirect corrosion sensors [19]. As shown in Figure 1, the direct corrosion sensors monitor corrosion process/rates directly due to various corrosion causes and corrosive environments. The indirect corrosion sensors monitor corrosion through corrosion causes (e.g., low pH, water, CO_2_) or consequences (e.g., wall thickness changes, leak vibration, strain change). Comprehensive understanding of corrosion processes from causes to consequences inspires corrosion sensor development strategies by identifying parameters or phenomena of interest as sensing targets. 

In this article, we briefly review and summarize conventional corrosion sensors which are well known, commonly used, and commercially available to provide the baseline and common practices in corrosion monitoring for the O&G industry. The main focus is emerging corrosion sensors including most recent technologies which are still in research and development (R&D) and technical transfer stages or only commercially available within the last two decades.

## 2. Conventional Corrosion Sensors

Conventional and commonly used corrosion sensors and SHM techniques in the O&G industry are discussed in this section. A review or summary on corrosion monitoring techniques in general or other areas is also available in References [19,20,21,22].

### 2.1. Corrosion Coupon

**Figure 2:**
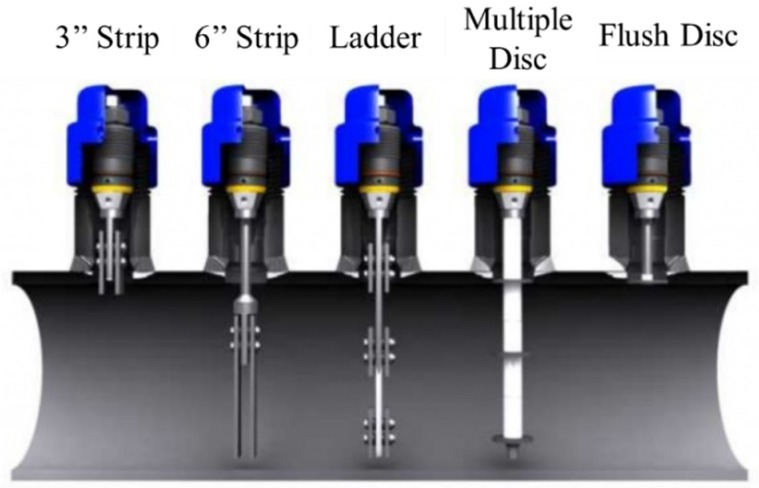
Corrosion coupons installed in the pipelines. The coupons are shown with a 90° axial rotation from the normal angle [24] (Courtesy of Rohrback Cosasco Systems).

Corrosion coupon weight loss measurement is the most well-established and longest-used method in industry to measure corrosion rates. Weight loss measurement is still held as the gold standard to evaluate corrosion rates before a variety of corrosion monitoring technologies. The working principle is that a corrosion coupon, made of a material of interest with designed weight and shape, is installed and exposed to the corrosive environment for a duration of time and then retrieved for after-corrosion weight measurement and inspection on the corroded coupon [23]. The corrosion coupon method is commonly used because of its simple working principle, easy operation, and versatility in material and shape (Figure 2). However, installation, removal, and after-corrosion lab analysis of the coupons require an extended time period. The corrosion coupons only provide an average corrosion rate during a certain period without real-time information, and they are point sensors with limited sensing coverage for O&G infrastructures.

### 2.2. Electrical Resistance Probe

**Figure 3:**
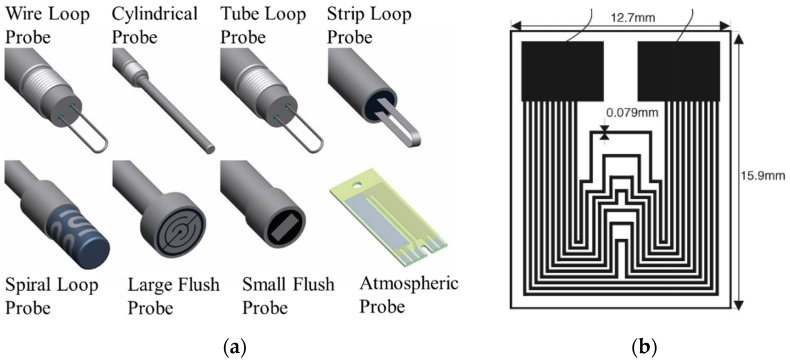
(**a**) Commercial electrical resistance (ER) sensing elements with different shapes [25] (Courtesy of Metal Samples Company); and (**b**) ER sensor with a multiple-line pattern of steel thin film which is sensitive to localized corrosion (Reprinted from Reference [26] with permission from Elsevier).

An electrical resistance (ER) probe is a commonly used approach for online corrosion rate monitoring with the capability of automatic and remote data logging in some advanced versions. It can be viewed as the “electrical” corrosion coupon that can be monitored in real-time via electrical resistance. Mass loss in the exposed metallic materials leads to an increase in electrical resistance. The exposed sensing element can be customized in material and shape for each specific application. ER probes work for both conductive media (e.g., water or oil systems with high water cuts) and non-conductive environments (e.g., oil, gas, and atmosphere). Some commercial ER probes are shown in Figure 3a [25]. A limitation of common ER probes is that they allow only the measurement of uniform corrosion, but Li et al. reported a multiple-line design of steel thin film ER probe which was sensitive to localized corrosion (Figure 3b) [26]. However, ER probes are still point sensors only capable of monitoring certain locations. An increase in sensing locations means an increase in total cost. Similar to corrosion coupons, installation locations need to be specifically picked to maximize the effectiveness of ER probes. Selecting locations is usually based on experience and some uncommon locations can be easily omitted even with significant corrosion. The electrical-based measurement enables electronic data collection and logging, but it also makes the ER probes prone to common electronic problems, which require regular maintenance and replacement. Importantly, electrical-based sensors must follow the intrinsic electrical safety requirements in the presence of flammable oil and natural gas.

### 2.3. Electrochemical Sensors

**Figure 4:**
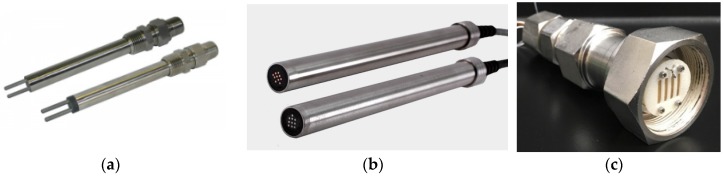
(**a**) Commercial 2-electrode linear polarization resistance (LPR) corrosion sensors [30] (Courtesy of Rohrback Cosasco Systems); (**b**) Coupled multi-electrode array corrosion sensors [33] (Courtesy of Corr Instruments, LLC); (**c**) Ion-conducting membrane-based advanced electrochemical sensor (AES) for simultaneous humidity and corrosion rate monitoring (Reprinted from Reference [35] with the permission of AIP Publishing).

Electrochemical sensors leverage the intrinsic electrochemical characteristics of corrosion and utilize electrochemical techniques such as galvanic current measurement, linear polarization resistance (LPR), electrochemical impedance spectroscopy (EIS), and electrochemical noise (EN) [22,27,28,29]. Advantages of electrochemical sensors include direct quantification of electrochemical corrosion rates and the capability of in-situ corrosion mechanism investigation with a variety of electrochemical techniques. LPR-based corrosion sensing is the most commercialized method among the electrochemical sensors because of relatively simple operation and data interpretation. For most of the commercial LPR probes (Figure 4a) [30], the electrodes (2 or 3) are often made of the same material instead of strictly following a classic electrochemical three-electrode system. The drawback of electrochemical sensors is that externally imposed potential or current may lead to an accelerated corrosion rate compared to the true value, so proper settings of electrochemical parameters (e.g., overpotential, scan rate, and Tafel slopes) and the electrode system design need to be carefully chosen. Additionally, the electrochemical sensors usually require an ion-conductive electrolyte, e.g., aqueous solutions, and they are not readily suitable for non-conductive environments without special modifications.

One commercially available electrochemical corrosion sensor to detect localized corrosion is based on the galvanic current in a coupled multi-electrode array [31,32], and the performance condition can reach 300 °C and 34.5 MPa (5000 psi) with proper packaging (Figure 4b) [33]. Also, water content and corrosion rates in simulated natural gas have been measured simultaneously using an ion-conducting membrane-based advanced electrochemical sensor (AES) (Figure 4c) [34,35]. Electrochemical sensors can also be designed to monitor pH and redox potentials in the environments.

### 2.4. Ultrasonic Testing Sensor

**Figure 5:**
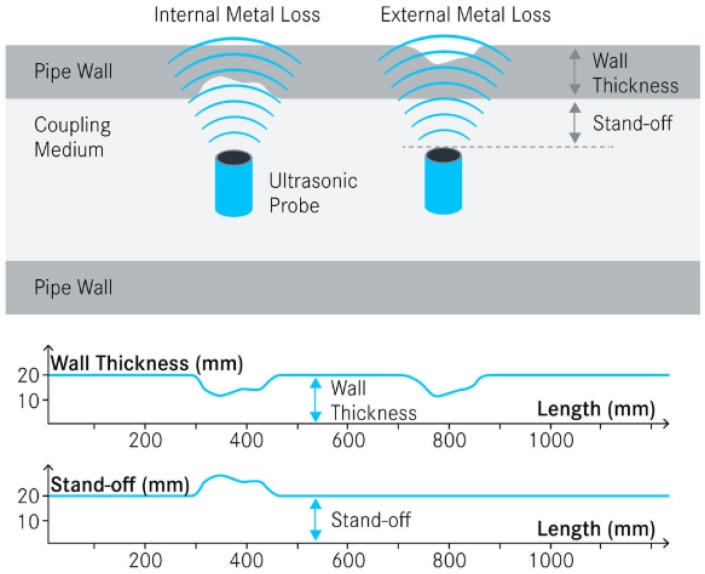
Schematic of the ultrasonic testing (UT) wall thickness measurements with capablities to discriminate internal and external mass loss [39] (Courtesy of NDT Global LLC).

Ultrasonic testing (UT) wall thickness measurement is one of the most popular nondestructive methods to monitor corrosion and structural health of pipes. A piezoelectric transducer generates high frequency (MHz) acoustic waves controlled through electric pulses, and these ultrasonic waves are emitted perpendicular to the pipe wall. The waves are bounced back by the external surface, inner surface, and geometric irregularities, which are received by the transducer. The tool measures the time interval between the arrivals of reflected echoes from inner and outer surfaces to calculate the wall thickness [36,37,38]. As shown in Figure 5 [39], the wall thickness information combined with the stand-off signal can differentiate the internal and external mass loss/flaw along the pipe. UT corrosion sensors have portable and fixed forms [29], and can also be integrated with in-line inspection devices. The UT method is capable of inspections with only one side accessible. The geometry resolution is related to the ultrasonic frequencies and often not sensitive enough to small features such as pitting corrosion or thin deposits. The acoustic-based sensors can be affected by dense highly attenuating muds and casing scales [3].

### 2.5. Magnetic Flux Leakage Method

**Figure 6:**
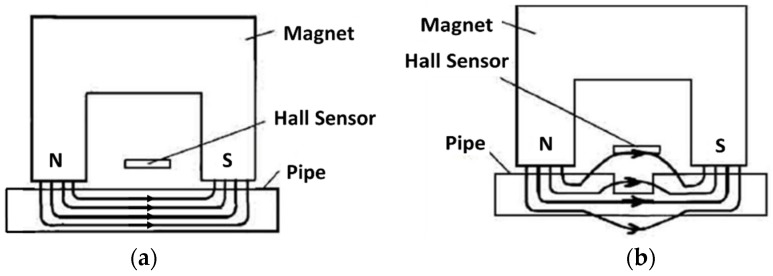
Principle of the magnetic flux leakage (MFL) method: (**a**) Magnetic flux lines mostly pass through the inside of ferromagnetic materials without defects, and (**b**) the Hall-effect sensor can detect the magnetic flux leakage when pipes have defects [41].

The magnetic flux leakage (MFL) method is a widely used nondestructive technology to detect anomalies in pipelines. The sensing principle is based on the magnetic properties of steels. When the ferromagnetic material is magnetized close to saturation under the applied magnetic field, the magnetic flux lines will mostly pass through the inside of the material when there are no defects, whereas the defect or corrosion sites will result in bending and leakage of magnetic flux lines [40]. The magnetic field is usually generated by an electromagnet, and a Hall-effect sensor is used to detect the magnetic flux leakage (Figure 6) [41]. The MFL method is good for large area inspection but it is limited for the material surface and near surface detection. Improvements are needed to determine the defect shapes and distinguish between internal and external defects [41].

### 2.6. Multi-Frequency Electromagnetic Sensors

**Figure 7:**
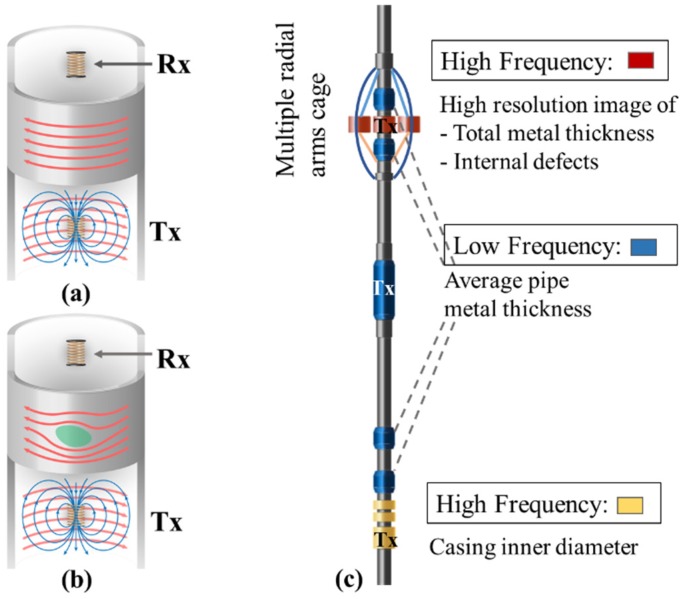
(**a**) Schematic of induced eddy currents (red arrows) in an uncorroded casing steel pipe with a solenoidal transmitter (Tx) and a receiver (Rx) within the pipe. The blue arrows represent the magnetic field lines around Tx. (**b**) Schematic of induced eddy currents flowing around a defect. (**c**) Schematic drawing of a multi-frequency electromagnetic (EM) tool for pipeline corrosion inspection: low-frequency transmitter (blue component labeled Tx) and receivers (other blue components) are used to measure average pipe metal thickness; one group of high-frequency transmitter (red component labeled Tx) and receivers (other red components) is used to measure high resolution images of total metal thickness and internal defects; and the other group of high-frequency transmitter (yellow component labeled Tx) and receivers (other yellow components) is used to measure the casing inner diameter [42].

Electromagnetic (EM)-based sensing provides another commonly used non-destructive corrosion monitoring technique. This method is based on the Faraday’s law of induction with many variations available. One example is the multi-frequency EM inspection sensor to detect corrosion and pipeline integrity. The sensor consists of a transmitter coil and a receiver coil. The transmitter coil is excited by an alternating current, and the generated alternating magnetic field induces eddy currents in the surrounding conductive pipes (Figure 7a). The primary EM field from the transmitter combined with a secondary field from eddy currents in the pipes induce a voltage in the separate receiver coil with a phase shift from the primary EM field [3,42,43]. The phase shift and magnitude change are related to the material electrical conductivity, magnetic permeability, and the presence of defects (Figure 7b). The pipe metal thickness can be computed from the low-frequency EM scan, and the high-frequency EM scans can discriminate the inner wall features due to the skin effect (Figure 7c). 

### 2.7. Pipeline Inspection Gauge

**Figure 8:**
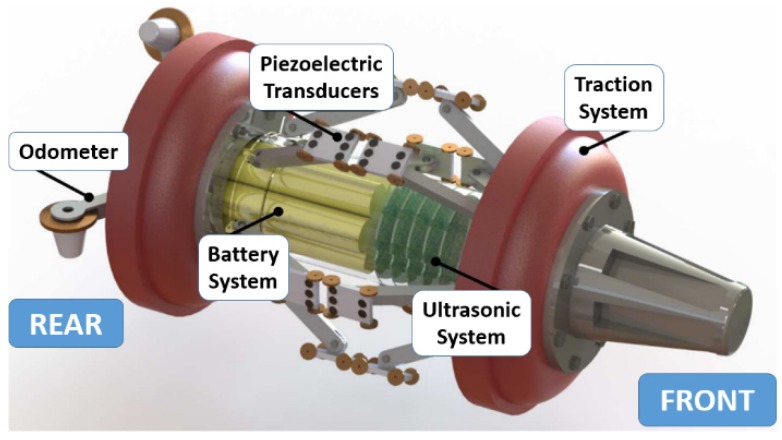
Schematic of a pipeline inspection gauge (PIG) integrated with ultrasonic sensors [36].

The commercially available pipeline inspection gauges (PIG) or in-line inspection tools (ILI) integrate a selection of sensors and cleaning tools. PIGs can be carried through the pipes by the flow of liquid or gas using the differential pressure while the pipelines are still operating, and they can travel and perform cleaning and inspections over a long distance. The equipped sensors such as UT sensors (Figure 8), MFL sensors, capacitive sensors, and EM sensors can collect data on corrosion, cracking, gouges, and anomalous weld seams [36]. For the inspection purpose, PIG is typically run every 5–7 years set by regulatory requirements or company policies [44,45]. The high cost associated with the PIG service is one main reason for low frequency of use. Despite comprehensive inspection, PIG cannot provide continuous monitoring of pipeline structural health. Therefore, cost-effective continuous corrosion sensors are in demand for the O&G infrastructures. 

All the sensors in this review are compared in Table 1 including the emerging technologies of optical fiber sensors and passive wireless sensors to be described in the following sections.

## 3. Emerging Corrosion Sensing Technologies

### 3.1. Optical Fiber Sensors

Optical fiber sensors (OFS) have emerged in recent years because of advantages such as nondestructive monitoring, in-situ distributive measurements, long reach, small size, flexibility, geometric versatility, light weight, inherent immunity to electromagnetic interference (EMI), compatibility to optical fiber data communication systems, and improved safety in the presence of flammables compared to electrical-based sensors [46,47,48]. The availability of cost-effective optical fibers and rapid advancement in OFS have stimulated the adoption of OFS in the O&G industry. Reviews on OFS for environmental, chemical, and H_2_ sensing are already available [47,49,50]. Here, we focus on OFS for corrosion and structural health monitoring in the O&G relevant applications.

According to the spatial distribution of measurements, OFS can be classified as point, quasi-distributed, and distributed. Point sensors monitor corrosion at discrete points, which are assessed by separate channels, i.e., each sensor only detects one point. A quasi-distributed sensor can monitor corrosion at multiple discrete points situated in a single optical channel. Distributed sensing can monitor the parameters continuously along the whole optical fiber with a specific spatial resolution by interrogating the continuously backscattered light [48].

#### 3.1.1. Point OFS for Corrosion

**Figure 9:**
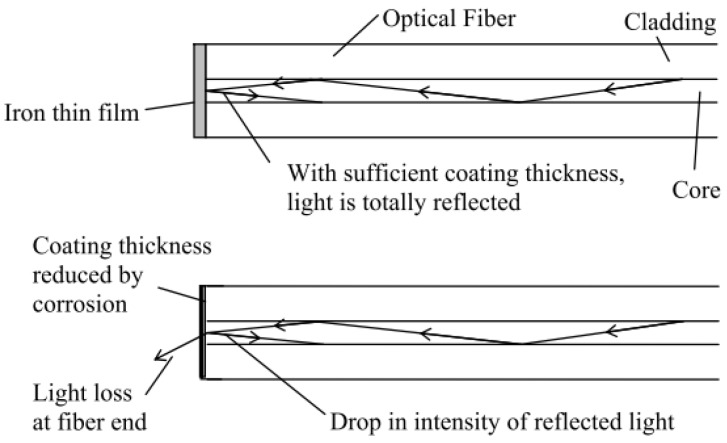
Point optical fiber sensor for corrosion monitoring based on reflected light from the Fe thin film coating [52].

Point corrosion OFS can be considered as the optical version of corrosion coupons. The point OFS usually have a sensing layer coated at one end or one section of optical fibers such as metallic films. The sensing principle of metallic film-coated OFS is based on interactions between photons and electronic structure of the metal in form of light reflection and absorption at the metallic films. As shown in Figure 9, when the metallic film (Fe or Fe–C alloy film) corrodes at the fiber end, the reflected light decreases, which can be detected at the other end of the optical fiber [51,52]. Alternatively, a Fe–C film is coated on a section of the fiber core, and the light transmission along the optical fiber increases as Fe is dissolved/corroded [53]. The corrosion-induced optical response can transmit through the optical fiber for a long distance, but point sensors only provide information at selected locations. 

**Figure 10:**
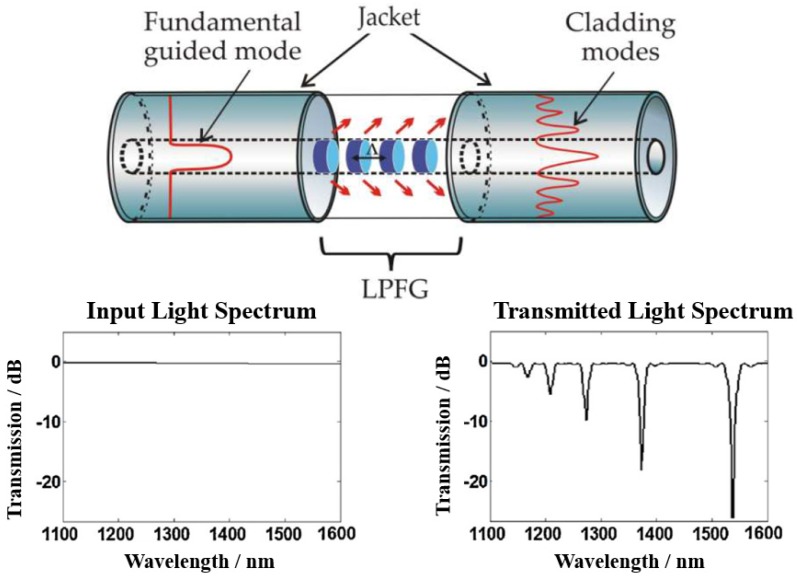
Schematic of long-period grating (LPG) structure [79] and an example of transmitted light spectrum after passing through LPG (Reprinted from Reference [80] with permission from Elsevier).

The long-period grating (LPG) provides another design of point sensor allowing light interaction with surrounding medium through the cladding modes. LPG has a typical periodicity (Λ) from 100 µm to 1000 µm, which is longer than that of the fiber Bragg grating (FBG), and couples light from a guided mode in the core into forward propagating cladding modes at certain wavelengths, resulting in dips in the transmission spectrum (Figure 10). These resonant wavelengths (λ_R_) can be expressed as in Equation (6) in terms of the periodicity and the difference between effective refractive indices of the fiber core and cladding modes [54].

λ_R_ = (n_eff,co −_ n_eff,cl,m_) Λ (6)

where n_eff,co_ and n_eff,cl,m_ are the effective refractive indices of the core and cladding modes, respectively. The resonant wavelengths are sensitive to changes in temperature, strain, or effective refractive indices of the cladding modes [55,56]. The cladding modes enable external environmental monitoring such as chemical or corrosion sensing. Compared with FBG, LPG shows almost an order of magnitude higher spectral shifts and is easier to fabricate due to the longer periodicity [55]. A multiplex of LPGs has been envisaged with different Λ and/or effective indices along the same fiber for quasi-distributed sensing; however, the multiple resonance peaks and large bandwidth of LPGs limit the multiplexing capabilities. Therefore, LPG sensors are mostly considered as point sensors. Based on the LPG sensitivity to the cladding mode refractive indices, a LPG fiber with a micro-layer of nano-iron and silica particles coated on the cladding has been demonstrated to monitor corrosion as the layer thickness and the sizes of iron particles get smaller in corrosive environments [57,58]. The LPG sensor with an electroplated Fe–C coating has been studied to monitor corrosion-induced mass loss [59,60]. LPG sensors can also monitor environmental parameters related to corrosion such as pH [61,62,63,64], humidity [65,66,67,68], CO_2_ [69,70,71], H_2_S [72,73], Cl^−^ and salinity [74,75,76,77]. More details on LPG based sensors can be found in References [56,78]. 

#### 3.1.2. Quasi-Distributed OFS for Corrosion

**Figure 11:**
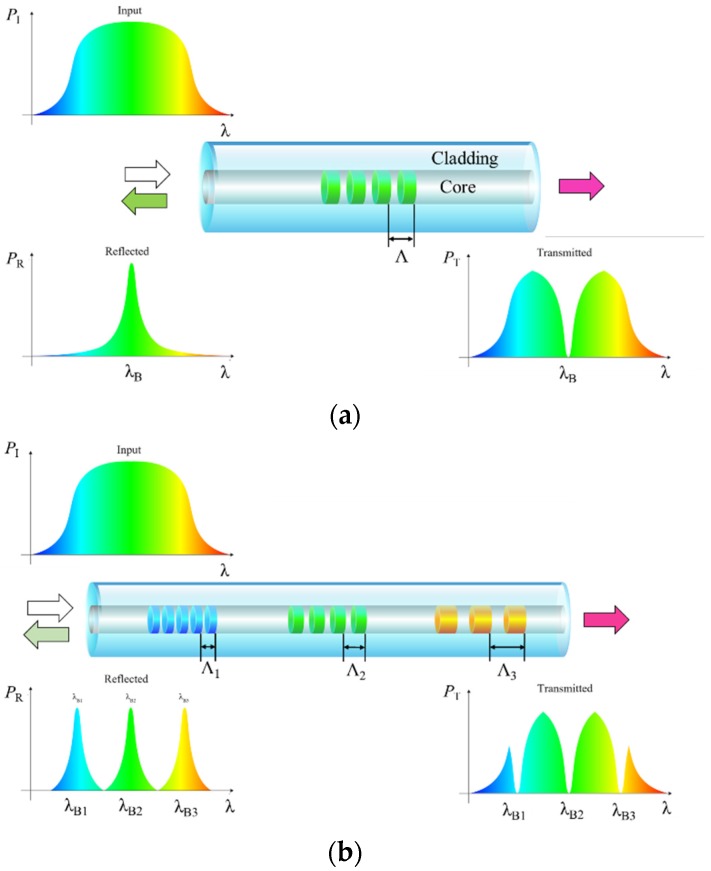
Schematic of (**a**) one fiber Bragg grating (FBG) and optical spectral responses [47] and (**b**) multiple FBG structures along an optical fiber and optical spectral responses [48].

Fiber Bragg grating-based sensors can be point or quasi-distributed sensors. As shown in Figure 11a, periodic gratings (periodicity Λ) along the core of an optical fiber lead to reflection of a certain wavelength (Bragg wavelength, λ_B_) and transmission of other wavelengths, following Equation (7) [47]. The environmental changes such as temperature and strain changes will cause shifting in the Bragg wavelength for each FBG sensor. Based on this principle, FBG-based sensors are capable of monitoring changes in parameters (e.g., temperature and strain) associated with corrosion in pipelines and wellbores. Several FBGs can be written along the same fiber with different Λ and/or effective indices (n_eff_), leading to several different Bragg wavelengths that can be spatially resolved along the optical fiber based on the reflected or transmitted spectrum (Figure 11b), enabling quasi-distributed sensing [48,81].

λ_B_ = 2 n_eff_ Λ (7)

**Figure 12:**
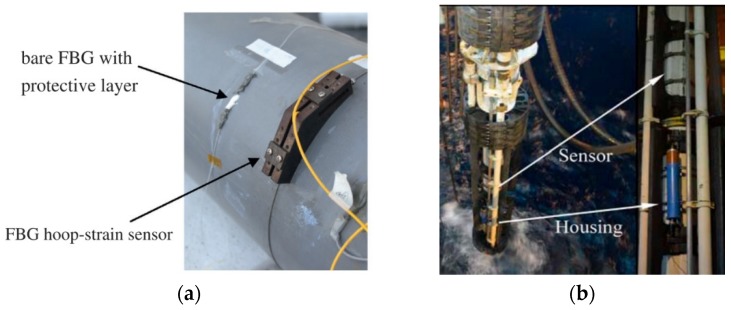
(**a**) Photo of FBG hoop strain sensors wrapping around a pipe (Reprinted from Reference [84] with permission from Elsevier); (**b**) Field demonstration of FBG-based sensors to monitor the riser stress for subsea drilling and operations [88].

FBG-based pressure sensors can be used to locate the pipeline leak based on the negative pressure wave (NPW) method. The onset of pipeline leak induces a pressure drop which propagates in both directions from the leak location, and the induced NPW will reach the FBG pressure sensors mounted on the pipe with time recorded through which the leak location can be computed [82,83]. The FBG-based strain sensors have been used to monitor the hoop strain (circumferential strain) of pipelines as an indicator of inner pressure fluctuation and the wall thickness reduction of pipelines (Figure 12a) [84,85,86]. It has also been reported that the FBG-based strain sensors were bonded directly on the surface of steel pipelines or inserted in the epoxy composite layers to measure the strain of repaired pipes [87]. Moreover, the FBG-based sensors have been installed onto the risers in field tests to demonstrate monitoring of the riser stress for subsea drilling and operations (Figure 12b) [88,89,90]. When coated with pH responsive hydrogel or hygroscopic polymers, FBG structures can also be used as pH or water sensors due to the mechanical expansion of the hydrogel or polymers [91,92,93].

#### 3.1.3. Distributed OFS for Physical Sensing

**Figure 13:**
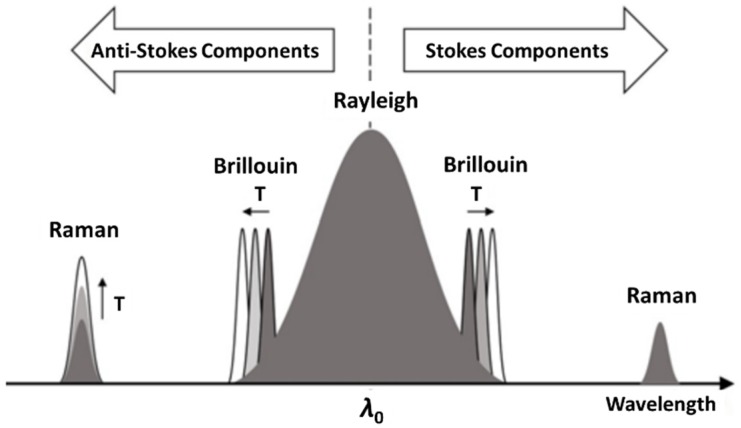
Three kinds of backscattered optical signals and their responses to temperature changes. (Reprinted by permission from Springer Nature: A review on optical fiber sensors for environmental monitoring, H. Joe, et al, 2018 [47]).

Distributed monitoring is a significant capability of OFS technology and is particularly suitable for long-distance infrastructures in the O&G industry such as thousands of miles of transmission pipelines. Compared to point sensors, distributed OFS enable continuous real-time monitoring over the whole structure with reduced cost per unit of length. Distributed OFS are based on the light backscattering at discontinuities along the optical fiber. There are three kinds of scattering: Rayleigh, Brillouin, and Raman scattering [47]. As shown in Figure 13, Rayleigh scattering is an elastic scattering caused by local fluctuations of the refractive index and is sensitive to surrounding changes such as temperature, strain/bending, and vibration. Brillouin scattering is an inelastic scattering caused by interaction with acoustic waves from lattice vibration and is sensitive to local temperature, strains, and deformation of the optical fiber. Raman scattering is another inelastic scattering caused by energy exchanges with molecular vibrations of the fiber. The anti-Stokes Raman scattering responds to the temperature changes whereas the Stokes Raman scattering is insensitive; therefore, the ratio of Stokes to anti-Stokes Raman scattering can be used to measure temperature [47,94]. The optical signals can be interrogated using Optical Time-Domain Reflectometry (OTDR) or Optical Frequency-Domain Reflectometry (OFDR) to realize spatially distributed monitoring. A detailed comparison on distributed interrogation techniques in terms of principle, resolution, limitation, and sensitivity can be found in Reference [95], including Brillouin OTDR, Raman OTDR, Rayleigh OFDR, etc. 

**Figure 14:**
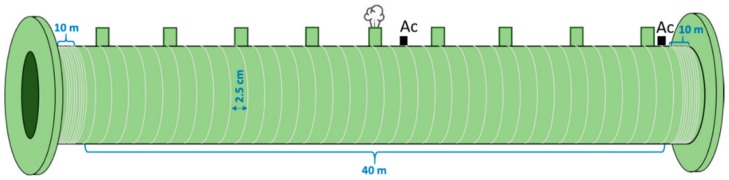
Schematic illustration of the employed fiber helically wrapping around the pipe to monitor leak-induced vibrations based on distributed acoustic sensing (DAS) [99].

Distributed temperature, strain, and acoustic sensing (DTS, DSS, and DAS) have been developed and matured over the last three decades [96], and they have been adopted for corrosion and structural health monitoring through investigations and field tests for the O&G industry. Besides monitoring temperature, strain, and vibration for well logging during O&G exploration [94], distributed OFS have been leveraged to monitor physical parameters related to corrosion, failure, and leak detection. DTS used for leak detection is based on the thermal signatures of the flowing products inside the pipelines. Heating transportation is one way to reduce the viscosity to efficiently flow highly viscous crude oil in the pipes, and the leak of heated oil results in a temperature change outside the pipelines, which can be detected through DTS [97]. Due to the Joule-Thomson effect, the leak of high pressure gas decreases the temperature and the leak of liquids increases the temperature, which allows DTS to detect pipeline leaks [94]. OFDR-based DSS has been studied to monitor wall thickness variation induced by internal corrosion based on the hoop strain monitoring using optical fibers around the pipes [86,98]. DAS, using coherent Rayleigh backscattering, has been investigated for detection of the leak-induced pipeline vibrations, either negative pressure waves or broadband leak-induced acoustic noises, using optical fibers helically wound around the pipes (Figure 14) [99,100]. Importantly, for long-distance monitoring, the sensing range of phase-sensitive OTDR has been demonstrated to be possible for extension to 131.5 km to monitor intrusion in pipelines [101].

#### 3.1.4. Distributed OFS for Chemical Sensing

Most of the sensors mentioned above are placed outside of the pipelines and measure corrosion indirectly through consequences of corrosion (e.g., temperature, strain, acoustic waves, leak vibration) after corrosion has occurred and the structural integrity is deteriorated; however, it is more ideal to monitor internal corrosion with sensors inside the pipelines and detect early corrosion onset before significant mass loss and structural integrity is compromised. 

**Figure 15:**
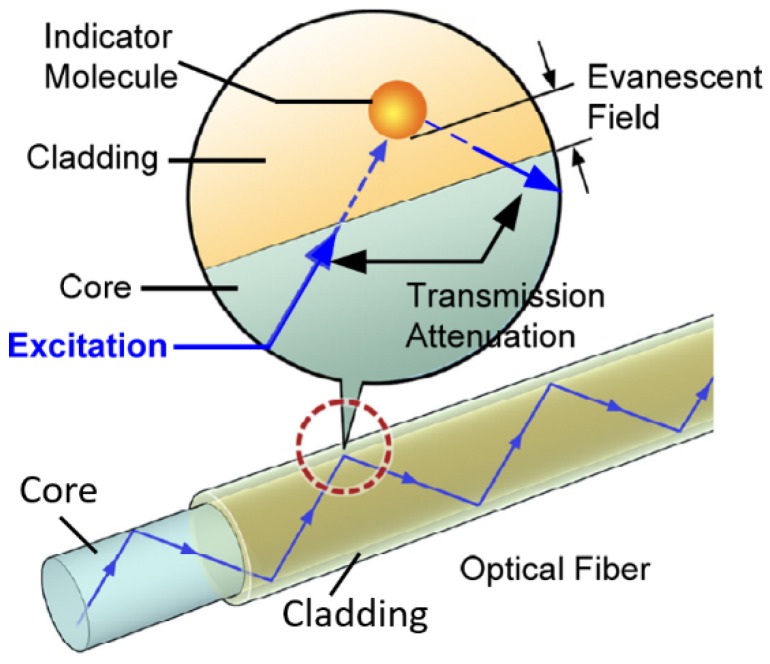
Schematic of evanescent field-based chemical sensor with a colorimetric indicator contained in the cladding of optical fiber [118].

Distributed chemical sensing (DCS) shows promising potential to monitor corrosive environments (Figure 1) before or upon early corrosion onset to facilitate corrosion mitigation, although DCS is less mature compared to DTS, DSS, and DAS. Optical fiber-based chemical sensors are enabled by functional chemical sensing coatings mostly on the fiber core or cladding, such as metallic films [53,102], oxides [103,104,105,106,107,108,109], polymers [49,110,111,112], nanomaterials [113,114,115], and metal-organic frameworks (MOFs) [116,117]. Figure 15 illustrates one example of the evanescent field-based chemical sensor [118]. The optical fibers can be etched, tapered, spliced, or side-polished to allow the light interactions with chemical sensing layers on the core or cladding and with the surrounding media [47,50,119]. 

Microstructured optical fibers provide a new type of fiber structure for OFS with great potential for DCS since the inception in the 1990s [120,121,122]. These fibers are featured with air holes running parallel to the longitudinal axis along the entire fiber length. If the air holes are periodically arranged in the cladding matrix, they are also known as photonic crystal fibers (PCF) [120,123]. Hollow-core PCF and index-guided PCF sensors have been demonstrated to detect gases such as methane, H_2_S, CO_2_, and acetylene with high sensitivity (ppm level) through direct interaction of light with gases in the holes [124,125,126,127,128]. A suspended-core fiber sensor has been developed for Cl^−^ detection with a Cl^−^ sensitive fluorescent material filled in the holes [129]. PCF LPG sensors have also been studied for Cl^−^ and humidity monitoring [130,131]. A birefringent PCF sensor has been designed to detect corrosion product-induced expansion for corrosion onset monitoring in reinforced concrete [132]. More sensing applications can be found in References [133,134,135]. Although microstructured fibers offer high sensitivity and flexible fiber designs, mass production and commercialization are still limited, which require cost-effective long-distance fabrication of these fibers.

**Figure 16:**
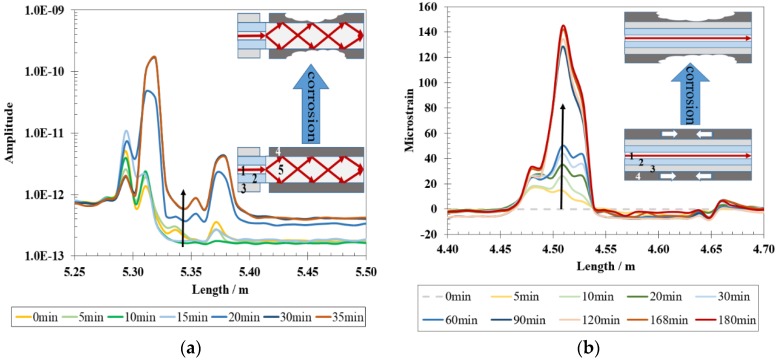
Metallic thin film coated optical fiber sensors (OFS) for distributed corrosion sensing interrogated using Optical Frequency-Domain Reflectometry (OFDR): (**a**) Rayleigh backscattered light increases as corrosion of Fe proceeds due to light absorption of metallic film; (**b**) Microstrain on the fiber increases with mass loss of coated Ni film due to release of compressive internal stress induced by Ni deposition [102]. Note: 1—single-mode fiber core; 2—cladding; 3—polymer jacket; 4—coated metallic film; 5—multi-mode fiber core.

A recent concept for corrosion detection involves the use of proxy materials integrated with the distributed OFS platform to monitor corrosion directly as a distributed optical “corrosion coupon” and provide insights into the corrosive conditions [5,6]. The corrosion-proxy distributed OFS is envisioned to be installed along the inner wall of pipelines to monitor internal corrosion. Metallic film-coated optical fibers have been demonstrated for distributed monitoring of corrosion when interrogated using OFDR. As shown in Figure 16, mass loss of metallic coating is monitored based on (a) intensity change or (b) strain change along the optical fiber [102,136]. The light intensity increases in the corroded region because the light absorption of metallic film decreases as the film becomes thinner. The increase in strain is caused by release of compressive internal stress induced by electroless deposition of Ni film.

**Figure 17:**
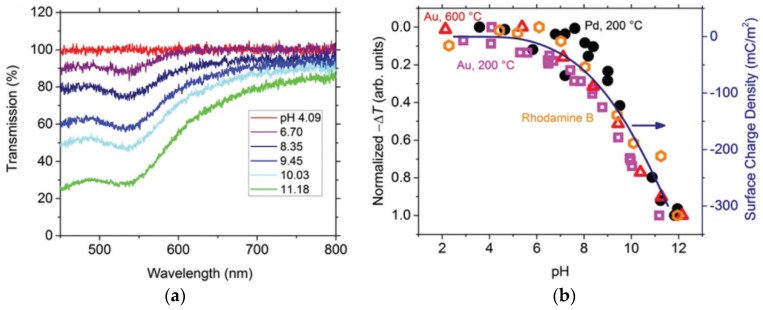
(**a**) Transmission spectra of optical fiber pH senor coated with localized surface plasmon resonance (LSPR) Au-nanoparticles incorporated SiO_2_ layer at different pH; and (**b**) pH sensing results from silica-matrix coatings embedded with a variety of optically active materials. (Reproduced from Reference [107] with permission from The Royal Society of Chemistry).

Environmental factors such as pH, water content, electrolyte conductivity, and acidic gas CO_2_ or H_2_S are critical for corrosion. Distributed sensing of these chemical parameters can determine the environmental corrosivity and therefore indirectly monitor corrosion. Although DCS has only been demonstrated in a few studies [102,137,138,139], the chemical sensing materials investigated for a broader range of fiber optics applications could be potentially utilized for the development of DCS to monitor corrosive environmental factors. There is a variety of pH sensing materials for integration with OFS, including localized surface plasmon resonance (LSPR) Au or Ag nanoparticles (NP) incorporated composites (Figure 17a) [107,140,141], organic dyes [142,143,144,145,146,147], fluorescent molecules [148,149,150,151,152], polymers [153,154,155,156], pH-sensitive hydrogel [157,158,159,160], etc. For the silica matrix coating, the surface charge density of silica matrix was found to correlate with the solution pH regardless of incorporated materials in the matrix layer (Figure 17b) [107,161]. Optical fiber pH sensors and pH sensitive materials are reviewed in more detail in References [160,162,163].

**Figure 18:**
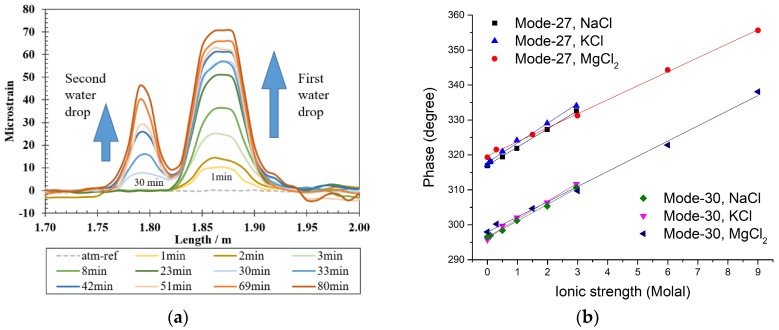
(**a**) Demonstration of distributed water detection in air based on the swelling-induced strain changes interrogated with an optical backscatter reflectometer (OBR). The first water drop was added at 1 min and the second water drop was added at 30 min [138]; (**b**) Phase shift-based optical fiber sensor (OFS) without any additional coating for simultaneous multi-parameter monitoring including ionic strength as a corrosivity indicator [119].

Water and salinity can also be monitored by OFS. Water condensation and presence have been detected by a fully distributed water sensor based on the hygroscopic property of the intrinsic polymer jacket of a commercial single-mode (SM) fiber, and the swelling-induced strain changes are spatially interrogated using OFDR (Figure 18a) [138,164]. The coatings of graphene oxide film and polymers (e.g., polyimide) have also been studied for water or humidity monitoring [137,165,166]. A multi-parameter OFS has been developed to detect the water/solution presence, ionic strength, and temperature simultaneously without any coating through analyzing phase shifts in all the modes (Figure 18b), and a sensor network can be designed for internal corrosion monitoring of natural gas transmission pipelines [119]. OFS for Cl^−^ and salinity monitoring are mostly based on the refractive index changes detected using tapered optical fiber, U-shaped fiber, SPR coating, or fluorescence sensitive material for chloride [129,167,168,169].

For acidic gas monitoring, gas sensitive coatings or gas absorption layers are often used in OFS. CO_2_-absorbing MOFs have been studied for CO_2_ monitoring and demonstrated quick and reversible responses [117,170,171]. Because dissolved CO_2_ can reduce the solution pH, CO_2_ sensors also employ pH indicators (colorimetric or fluorescent dyes) within various sensing layers such as silica gel coating, polymer matrix with quantum dots, and sol–gel matrix with silica nanoparticles [172,173,174,175,176,177]. H_2_S monitoring often utilizes reactive sensing materials such as Ag [178,179], Cu [180,181], ZnO [182,183], CuO doped SnO_2_ [184], CdO [185], and fluorescent or luminescent indicators [186,187,188]. More H_2_S sensitive materials can be found in References [189,190]. 

Table 2 lists some examples of OFS chemical sensing layers for corrosivity monitoring.

#### 3.1.5. Challenges of OFS Application in the O&G Industry

HTHP in the O&G wellbores impose a big challenge on downhole monitoring with harsh conditions due to CO_2_, H_2_S, and mechanical stress. The downhole temperature is commonly 150–200 °C, and it can reach as high as 300 °C in some cases [94]. Although OFS possess advantages for downhole sensing (e.g., thermally and chemically stable, small size, light weight, long reach, and no electronics required downhole), silica fibers can suffer from long-term instability and hydrogen darkening due to hydrogen ingress when exposed to hydrogen/water especially at high temperature, thereby dramatically reducing their rated operational temperatures for long-term deployment. The formation of the Si–H bond and adsorption of OH^−^ ions cause the extrinsic attenuation along the fiber [195]. A hermetic carbon layer can be added between the cladding and the polymer jacket to protect silica fibers against hydrogen induced attenuation, but this specialty fiber is only rated up to 200 °C [196]. Therefore, protective coatings or proper OFS designs are paramount for HTHP sensing. Alternative fibers such as sapphire fibers can be suitable for extreme high temperature sensing (up to 1800 °C) if the cost and cladding challenges can be overcome and fibers can be produced at sufficient lengths to be relevant for the O&G applications [197].

Distributed interrogation with high resolution over long distances is another challenge for the long-distance O&G infrastructures such as hundreds of thousands of miles of gathering and transmission pipelines. Meanwhile, low-cost interrogation system and effective deployment of optical fiber sensors in the O&G infrastructures are also critical to make OFS more competitive than existing corrosion monitoring technologies. Phase-sensitive OTDR and Brillouin optical time-domain analysis (BOTDA) are promising interrogation approaches for monitoring >100 km distance [100,101,198,199,200]. There is often a trade-off between the interrogation distance and the spatial resolution. For distributed physical sensing, cross-sensitivity between multiple parameters requires discrimination from one another, e.g., T and strain effects [201,202,203,204]. For distributed chemical sensing, most chemical sensing layers require “leakage” of light from the fiber core, resulting in increased loss of light power and therefore limiting the interrogation distance.

### 3.2. Passsive Wireless Sensors

Passive wireless sensors constitute another emerging technology for structural health monitoring, which do not require active source of energy or active electronics at the sensing location and can wirelessly transfer energy and signals. Elimination of local batteries, active electronics, and electrical wiring is critical to improve sensor stability and durability at HTHP and harsh environments and to make sensors more compatible with moving parts. The wireless feature also makes it possible for monitoring in inaccessible areas. Due to the small size and low cost, passive wireless sensors can be deployed ubiquitously in the system of interest. Passive wireless sensors for corrosion and SHM are mostly based on the passive radio-frequency identification (RFID) and the surface acoustic wave (SAW) techniques [205,206,207,208].

#### 3.2.1. Passive Radio-Frequency Identification Sensors

**Figure 19:**
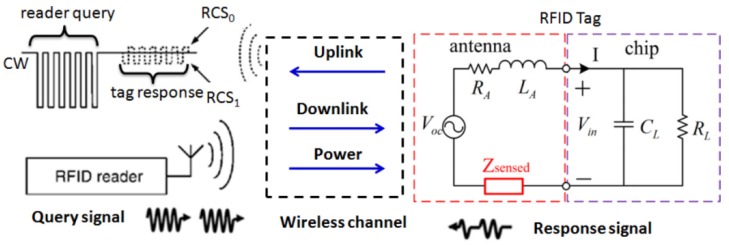
Schematic of a passive radio-frequency identification (RFID) antenna sensor system including a RFID tag, a reader, and antennas [209].

Passive RFID sensors form a large group of passive wireless sensors for corrosion and SHM, especially the chipless RFID sensors, and they have advantages of low cost, light weight, small size, and wireless remote sensing [206,207]. RFID technology usually consists of three components: a small tag unit (or transponder), a reader (or transceiver), and antennas. Figure 19 shows one example of a passive RFID antenna sensor system [209]. Passive RFID tags receive RF signals from the reader and respond with identity and sensing signals through the antennas [210]. Different from chip-based RFID tags, chipless RFID tags do not have an onboard silicon chip on the circuitry. Chipless RFID sensors categorically have three types: time-domain reflectometry (TDR)-based; frequency modulation-based, and phase-encoded chipless RFID sensors [211]. SAW devices can also be designed to be RFID tags [205,212,213].

RFID sensors can directly monitor corrosion when incorporated with corrosion sensitive proxy materials in the sensor configuration. With a corrosion sensitive link or connector (e.g., metal or steel of interest) between the circuit and antennas on a RFID sensor, the circuit will not be properly energized through antennas to respond back due to corrosion of the link, which indicates occurrence of corrosion [214]. In another design, the EM shielding effect of metallic materials between the reader and the RFID tag is exploited where the plastic-packaged RFID tags are coated/covered with metallic materials or metal-filled conductive paint. When exposed to corrosive environments, degradation of the coating dampens its EM shielding effect and improves communication between the reader and the tag [215,216]. 

Moreover, corrosion and structural health can be indirectly monitored with RFID sensors. An LC resonator on a passive tag with an interdigitated capacitor has been studied to monitor the coating lift-off from pipelines and water ingress [217]. Defects and cracking progression have been detected using low frequency or (ultra) high frequency RFID antenna-based sensors [207,209,218,219]. Corrosion potential, chloride ion concentration, and pH have been measured using chip-based RFID sensors with integrated sensing electrodes [220,221,222,223]. Chemical sensing functionality can be achieved with suitable films on the RFID tags to monitor, for example, CO_2_, H_2_S, humidity and pH [224,225,226]. Sun et al. have demonstrated an innovative RFID corrosion sensor based on Events as Power Source (EPS) where the corrosion process is monitored as an event while powering the wireless sensor [227]. The micro-energy produced by the electrochemical reactions during corrosion is harvested through a supercapacitor-based chip to power the sensor.

#### 3.2.2. Surface Acoustic Wave Sensors

**Figure 20:**
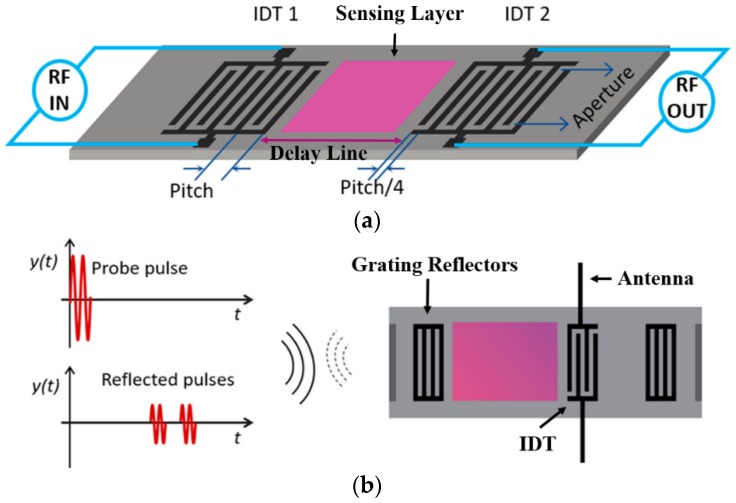
Schematic of (**a**) a surface acoustic wave (SAW) sensor with a coated sensing layer on the delay line and (**b**) a functionalized SAW sensor interrogated wirelessly [228,229].

SAW sensors are of particular interest as passive wireless sensors because of their small size, cost efficiency, ease of fabrication, compatibility with wireless telemetry, and adaptability to many applications (Figure 20). SAW devices consist of interdigitated transducers (IDTs) fabricated on a piezoelectric substrate. IDTs are periodic metallic electrodes (fingers) in the form of two combs intercrossing from opposite sides, and they can convert the RF signal to SAWs on piezoelectric surfaces and vice versa. There are variants of SAWs such as Rayleigh, shear horizontal SAW (SH-SAW), Love, Stoneley, Lamb, and Leaky waves that can be excited on piezoelectric substrates. When the emitting IDT is excited by an external RF signal, SAWs are launched on the piezoelectric substrate and propagate on the substrate surface and perpendicular to the IDT aperture. When the SAWs reach a second IDT, the waves can be converted to output RF signals (Figure 20a) or some waves get reflected back to the emitting IDT for output RF signals. Alternately, when the SAWs reach grating reflectors, they get reflected back to the emitting IDT for RF signal processing (Figure 20b) [228,229]. SAW-based sensing is accomplished by measuring changes in the phase velocity and/or amplitude of the waves caused by property changes in the propagation path such as temperature, mass, electrical, and mechanical changes; therefore, SAW sensors can be employed for monitoring many physical parameters (e.g., temperature [213,230,231,232], pressure [233,234], and strain [235,236]) as well as chemical species in the gaseous and aqueous phases. Detailed reviews on fundamentals of sensing mechanisms and applications can be found in References [228,229,237]. 

**Figure 21:**
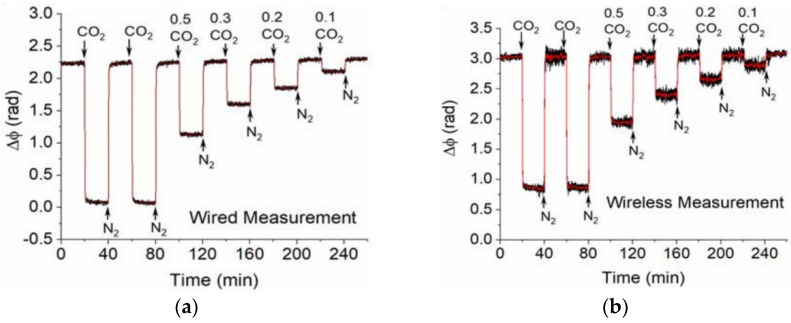
Responses of a surface acoustic wave (SAW) sensor coated with zeolitic imidazolate framework-8 (ZIF-8) metal-organic framework (MOF) film to various concentrations of CO_2_ through wired or wireless measurements (© 2018 IEEE. Reprinted with permission from Reference [238]).

SAW chemical sensors are usually coated with target-specific chemical-sensitive materials such as polymers, MOFs, metals, and metal oxides (Figure 20). For sensing in the gaseous phase, Rayleigh waves are most commonly utilized with gas absorbing or reactive layers coated on the SAW devices as functional sensing layers [229]. Real-time monitoring of the O&G relevant gases such as CO_2_ and CH_4_ using SAW sensors coated with MOF materials has been demonstrated in wired and wireless operations (Figure 21) [116,238]. CO_2_ sensitive polymers or nanomaterials (e.g., graphene) have also been studied for CO_2_ SAW sensors [239,240,241]. H_2_S can also be monitored by SAW sensors coated with H_2_S sensitive films such as CuO, SnO_2_, Cu, and WO_3_ [242,243,244,245,246].

Application of SAW sensors in an aqueous medium for detection of corrosion onset or monitoring corrosion stimulants (Table 2) requires consideration of devices with specific wave modes such as SH-SAW [247,248], because not all SAW modes (e.g., the Rayleigh mode) can survive in the aqueous phase. The devices with appropriate SAW modes, when functionalized with specific sensing layers (e.g., Al, ZnO coating), can be adopted to monitor corrosion and chemical parameters that can cause corrosion, thereby leveraging them for the O&G applications [249,250,251,252]. Alternatively, a device with any SAW mode might be used to monitor corrosion when designed to avoid direct contact of propagating acoustic waves with liquids through proper packaging [253]. Additionally, challenges exist with wireless telemetry in the aqueous phase due to strong absorption of typical RF electromagnetic radiation by aqueous solutions. 

## 4. Summary and Outlook

The ability to monitor corrosion online before structural integrity is compromised can have a significant impact on preventing catastrophic events resulting from corrosion. Corrosion sensors for structural health monitoring in the O&G industry have been reviewed including conventional corrosion sensors and emerging sensor technologies in terms of sensor designs, advantages, and limitations. Corrosion sensors can be generally categorized into two types: direct and indirect corrosion sensors. Conventional corrosion sensors encompass corrosion coupons, electrical resistance probes, electrochemical sensors, ultrasonic testing sensors, magnetic flux leakage sensors, electromagnetic sensors, and pipeline inspection gauges. The emerging sensor technologies highlight optical fiber sensors and passive wireless sensors such as RFID and SAW sensors. 

Optical fiber sensors have the advantages of nondestructive monitoring, in-situ distributive measurements, long reach, small size, light weight, flexibility, inherent immunity to EMI, compatibility to optical fiber data communication systems, and improved safety in the presence of flammable gas/oil compared to electrical-based sensors. According to spatial distribution of the measurements, OFS can be classified as point, quasi-distributed, and distributed with different sensing principles and interrogation methods. Distributed monitoring enabled by the OFS technology is particularly suitable for long-distance infrastructures in the O&G industry such as transmission pipelines. DTS, DSS, and DAS have been developed and matured over the last three decades for physical parameter monitoring. As a less mature technology, DCS shows promising potential to detect early corrosion onset and monitor corrosive environments such as direct mass loss, pH, water, salinity, and acidic gases before or upon early corrosion onset and therefore facilitate corrosion mitigation. It is crucial to have effective deployment of optical fiber sensors in the O&G infrastructures with low-cost, long-distance, and high spatial resolution interrogation.

Passive wireless sensors have advantages of small size, cost efficiency, elimination of active power, ease of fabrication, compatibility with wireless telemetry, and adaptability to many applications. Elimination of local batteries, active electronics, and electrical wiring is critical to improve sensor stability and durability at HTHP and harsh environments and to make sensors more compatible with moving parts. Passive RFID sensors have been explored for corrosion and structural health monitoring with versatile designs. SAW sensors have been employed for monitoring many physical parameters (e.g., temperature, pressure, and strain) as well as chemical species in the gaseous and aqueous phases. Due to the small size and low cost, passive wireless sensors can be deployed ubiquitously in the system of interest. Main challenges exist with wireless telemetry in highly attenuating media such as aqueous or muddy conditions. 

Both emerging technologies are promising for continuous real-time in-situ corrosion monitoring and SHM of the O&G infrastructure. Additional R&D are required to develop and design chemical sensing materials with high sensitivity, selectivity and stability to integrate with the sensing platforms, especially for HTHP and harsh environments in the subsurface wells or other extreme conditions. 

## Disclaimer

This work was funded by the Department of Energy, National Energy Technology Laboratory, an agency of the United States Government, through a support contract with Leidos Research Support Team (LRST). Neither the United States Government nor any agency thereof, nor any of their employees, nor LRST, nor any of their employees, makes any warranty, expressed or implied, or assumes any legal liability or responsibility for the accuracy, completeness, or usefulness of any information, apparatus, product, or process disclosed, or represents that its use would not infringe privately owned rights. Reference herein to any specific commercial product, process, or service by trade name, trademark, manufacturer, or otherwise, does not necessarily constitute or imply its endorsement, recommendation, or favoring by the United States Government or any agency thereof. The views and opinions of authors expressed herein do not necessarily state or reflect those of the United States Government or any agency thereof.

## Figures and Tables

**Table 1:** Summary of different corrosion sensors for the oil and gas industry.

Sensor	Temporal	Spatial	Advantages	Disadvantages
Corrosion coupon	A few months	Point sensor	Gold standard, Simple, Easy to operate	General corrosion, Not real-time
Electrical resistance probe	Real-time	Point sensor	Real-time, Remote sensing compatible	Uniform corrosion, Electrical based
Electrochemical sensor	Real-time	Point sensor	Various in-situ electrochemical techniques	Electrical based, Mostly for conductive liquids
Ultrasonic sensor	Real-time	Point sensor, PIG	Non-intrusive	Not sensitive to small thin features
Magnetic flux leakage sensor	Real-time	Point sensor, PIG	Nondestructive	Limited for surface detection
Electromagnetic sensor	Real-time	Point sensor, PIG	Nondestructive, Inner wall features	Not sensitive to small defects
Pipeline inspection gauge	Every 5–7 years	Run through pipes	Comprehensive sensing/logging, Long distance	Costly, not frequent
Optical fiber sensors	Real-time	Distributed linear sensors	Distributed sensing for a long distance, Multi-parameter	Cost of interrogation instrument
Passive wireless sensors	Real-time	Ubiquitous point sensors	Small size, Passive, Wireless capability, Low cost	Wireless telemetry in attenuating media

**Table 2:** Chemical sensing parameters and examples of optical fiber sensing layers for corrosion monitoring.

Parameter	Sensing layer	Test Condition	Performance and Comments
Corrosion	Fe [136]	30 °C, 1 atm, CO_2_ saturated 3.5 wt.% NaCl	Distributed sensing, nm-scale mass loss sensitivity
	FeC [53]	0.18–1.8 mol/L H_2_SO_4_	10s of uW increase in light transmission in <10 min
	Al [191]	0.05 mol/L NaOH	uW increase in light transmission in 5 min
pH	Au-NP in SiO_2_ matrix [107]	Room temperature (RT) and 80 °C, 1 atm	pH 2–12, quick response
	Organic dyes in SiO_2_ matrix [143,146]	RT, 1 atm *	pH 3–12
	Polyaniline [154]	RT, 1 atm *	pH 2–12, >1 month stability in air
	fluorescent Poly(p-pyridiniumphenylene ethynylene)s [192]	RT, 1atm	pH 1–10
	pH-sensitive hydrogel [158]	RT, 1atm	Wavelength 1.94 nm/pH, pH 3–10
Water	Polyimide [137]	30 – 50 °C, 1atm	38.5 ± 1.9 microstrain/%Relative Humidity (RH)
	Graphene oxide film [165]	27–67 °C, 1atm	Wavelength 0.145–0.915 nm/%RH for 32–97.6% RH; Intensity 0.427 dB/%RH for 58.2–92.5% RH
Salinity or Cl^−^	SPR based Al/TiO_2_ [193]	RT, 1atm *	Accuracy of 0.1‰ salinity
	Fluorescent Lucigenin [129]	RT, 1atm *	Detection limit of 0.02 mol/L Cl^−^
CO_2_	Zeolitic imidazolate framework-8 (ZIF-8) MOF [170]	RT, 1atm	10s of seconds response, Reversible, Linear calibration
	Dyes (e.g., methyl red) in SiO_2_ gel [172]	15–60 °C, 1atm	2–3 seconds response
	Fluorescent dye HPTS (1-Hydroxypyrene-3,6,8-trisulfonic acid trisodium salt) [174,176]	5–35 °C, 1atm	Sol–gel matrix doped with silica particles improved sensitivity
H_2_S	Ag layer [178]	30 °C, 1 atm	90% transmittance drop in 15 minutes in 0.1 mol/L H_2_S solution
	CdO in porous SiO_2_ [185]	450 °C, 1atm	25–30 minutes response time for 1–100 ppm H_2_S, Irreversible but regenerable
	SPR based Ag/NiO doped indium tin oxide (ITO) [194]	RT, 1atm *	100 ppb–100 ppm H_2_S, Sensitivity decreased with H_2_S concentration

* Assumed at RT and 1atm when lack of clarification on test conditions.
